# Supplementary material for: Single-cell RNA sequencing reveals the fragility of male spermatogenic cells to Zika virus-induced complement activation
Source: Nat Commun. 2023 Apr 29;14:2476. doi: 10.1038/s41467-023-38223-z (PMC10148584; doi:10.1038/s41467-023-38223-z)
Supplement: Supplementary file 1 — Supplemental information [file 41467_2023_38223_MOESM1_ESM.pdf]

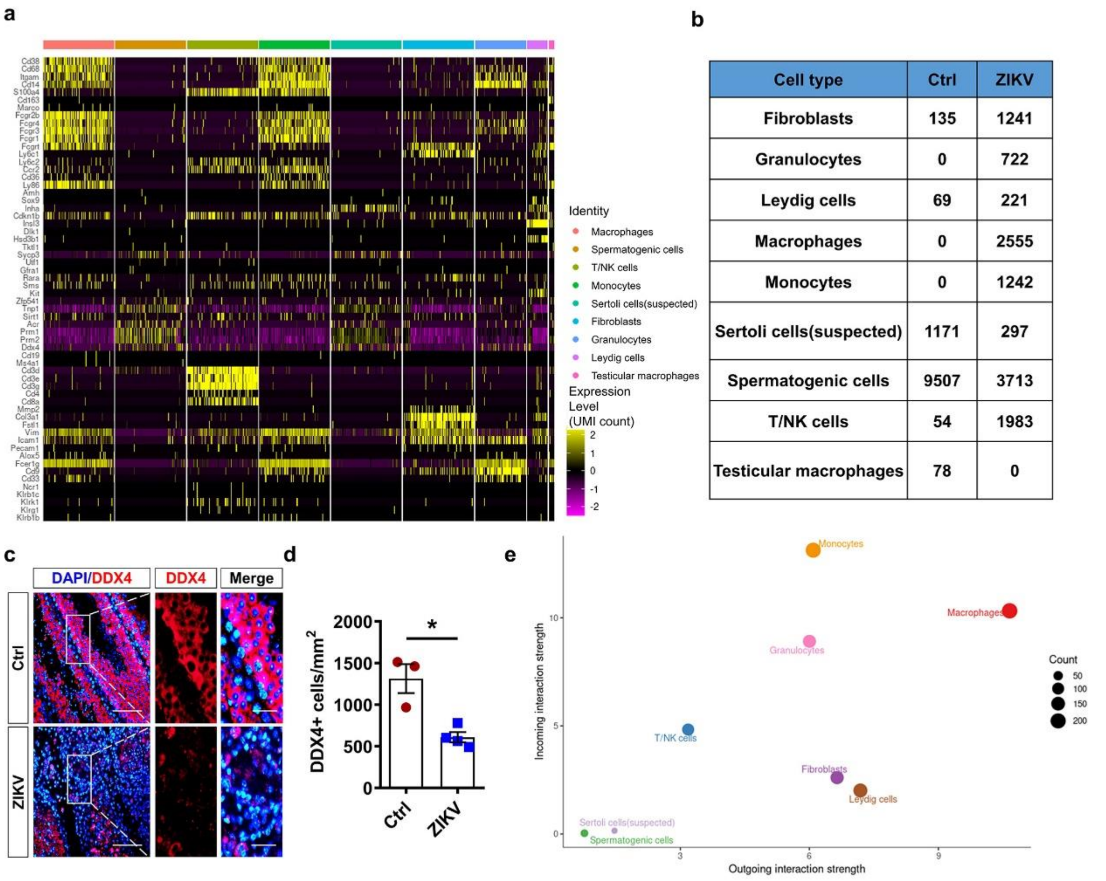

Cell type	Ctrl	ZIKV
Fibroblasts	135	1241
Granulocytes	0	722
Leydig cells	69	221
Macrophages	0	2555
Monocytes	0	1242
Sertoli cells(suspected)	1171	297
Spermatogenic cells	9507	3713
T/NK cells	54	1983
Testicular macrophages	78	0

Supplementary Fig S1. Cells clustering by single-cell sequencing after ZIKV infection.

Testes from PBS-injected (control) or ZIKV-infected A6 male mice were subjected to single-cell sequencing. **(a)** Cells from control or ZIKV-infected testes in each cluster were sorted into various cell types based on series cell markers (*Cd38*, *Cd68*, *Itgam*, *Cd14*, *S100a4*, *Cd163*, *Marco*, *Fcgr2b*, *Fcgr4*, *Fcgr3*, *Fcgr1*, *Fcgrt*, *Ly6c1*, *Ly6c2*, *Ccr2*, *Cd36*, *Ly86* for identification of **exogenous monocytes/macrophages and testicular macrophages**; *Amh*, *Sox9*, *Inha*, *Cdkn1b* for **Sertoli cells**; *Insl3*, *Dlk1*, *Hsd3b1* for **Leydig cells**; *Tktl1*, *Sycp3*, *Kit*, *Sms*, *Zfp541*, *Tnp1*, *Sirt1*, *Acr*, *Prm1*, *Prm2*, *Ddx4* for all **spermatogenic cells**; *Cd19*, *Ms4a1* for **B cells**; *Cd3d*, *Cd3e*, *Cd3g*, *Cd4*, *Cd8a*, *Ncr1*, *Klrb1c*, *Klrk1*, *Klrg1*, *Klrb1b* for **T/NK cells**; *Mmp2*, *Col3a1*, *Fstl1*, *Vim* for **fibroblasts**;

Icam1, *Pecam1* for **endothelial cells**; *Alox5*, *Fcer1g*, *Cd9*, *Cd33* for **granulocytes**). **(b)** The number of cells in all cell clusters in the testes from control and ZIKV-infected mice. **(c, d)** Immunofluorescence staining with anti-DDX4 antibody in testis sections from control or ZIKV-infected A6 mice at 14dpi **(c)**. Nuclei were visualized with DAPI. Scale bar, 25 μm (10 μm in enlarged panels). DDX4+ cells **(d)** were quantified and expressed as cells/mm^2. Results were shown as means ± SEM (n=3 mice for ctrl and 4 for ZIKV-infected) and analyzed using the two-sided Student's t test. *$p < 0.05$. **(e)** Scatter plot of the incoming/outgoing interaction strength of each cell clusters in ZIKV-infected testes. Exact p values in Source Data file. Source data are provided as a Source Data file.

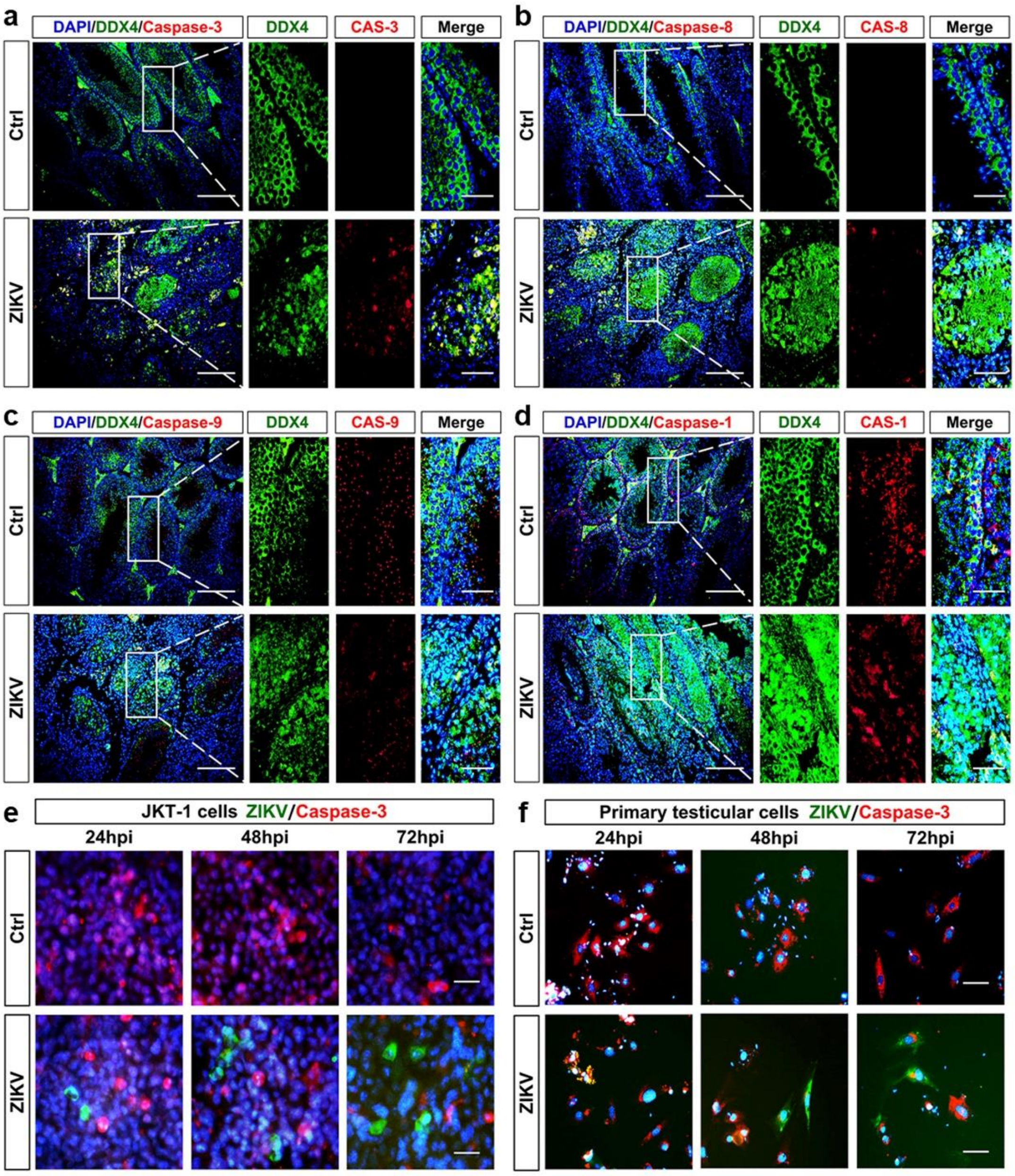

Supplementary Fig S2. The expression of genes related to known death pathways in *Ddx4*-expressing cells.

(a-d) Co-immunofluorescence staining of anti-DDX4 and anti-Caspase-3 antibody **(a)**, anti-Caspase-8 antibody **(b)**, anti-Caspase-9 antibody **(c)** or anti-Caspase-1 antibody **(d)** in testis sections of control or ZIKV-infected mice at 14dpi. Nuclei were stained with DAPI. Scale bar 25 µm (20 µm in enlarged panels). **(e, f)** Immunofluorescent staining of caspase-3 in ZIKV-infected JKT-1 cells **(e)** or primary testicular cells from A6 mice

(f) at 24-72hpi. Nuclei were stained with DAPI. Scale bar, 25 μm.

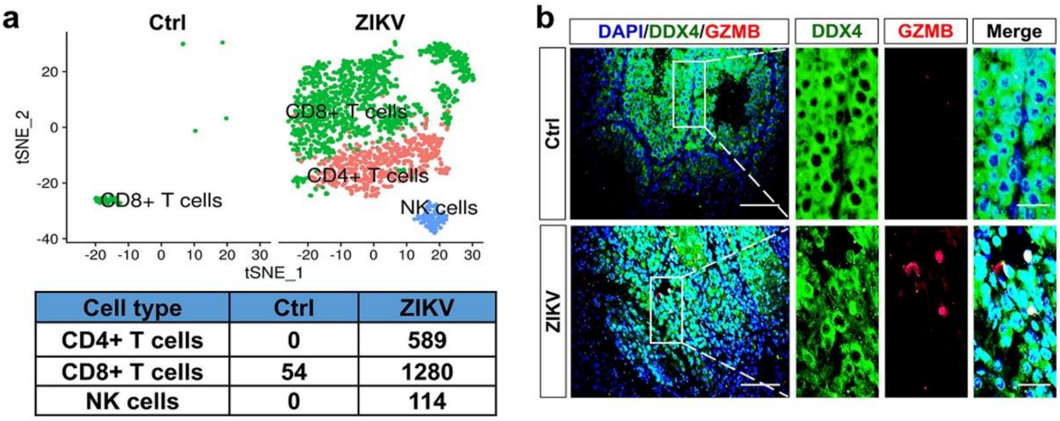

Cell type	Ctrl	ZIKV
CD4+ T cells	0	589
CD8+ T cells	54	1280
NK cells	0	114

Supplementary Fig S3. ZIKV-infected testicular cells did not undergo apoptosis and were not attacked by granzyme B.

(a) T cells in the testes from control or ZIKV-infected mice were sorted into CD8+ T cells, CD4+ T cells and NK cells subsets. The distribution of CD8+ T cells, CD4+ T cells and NK cells were shown in tSNE chart. **(b)** Co-immunofluorescence staining of DDX4 and GZMB in testes sections of control mice or ZIKV-infected A6 male mice at 14dpi. Nuclei were stained with DAPI. Scale bar 25 μm (10 μm in enlarged panels).

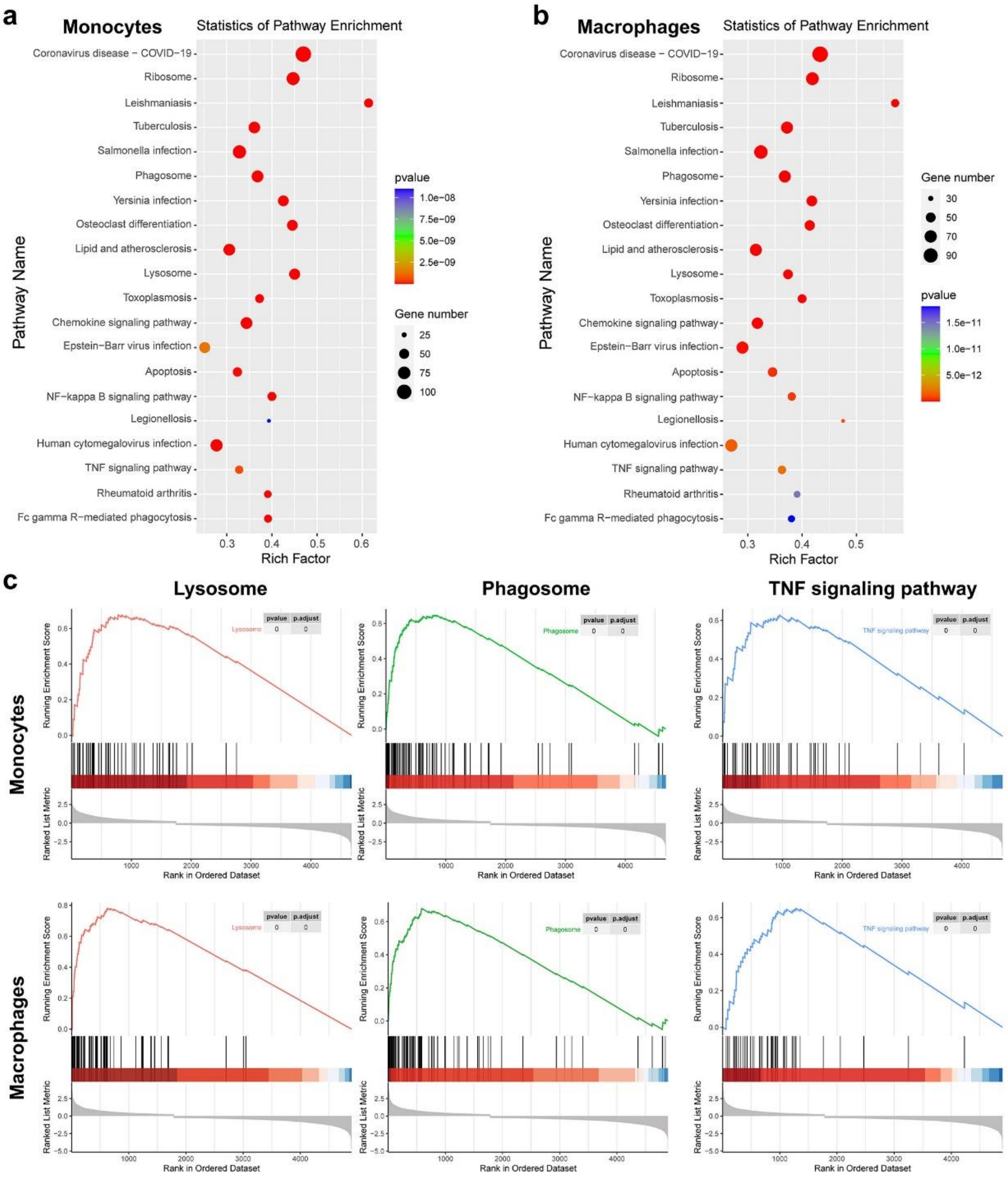

Supplementary Fig S4. Enrichment plots in monocytes and macrophages.

(a, b) The expression levels of up-regulated and down-regulated genes in exogenous monocytes **(a)**, and macrophages **(b)** were analyzed by KEGG enrichment analysis. Significance was examined by the two-sided hypergeometric test. **(c)** GSEA assay was performed in order to investigate the phagocytosis and inflammation related pathway genes of the exogenous monocytes and macrophages. 'Lysosome', 'Phagosome', 'TNF signaling pathway' were similarly enriched in monocytes and macrophages.

Significance of GSEA results was examined by the two-sided permutation test.

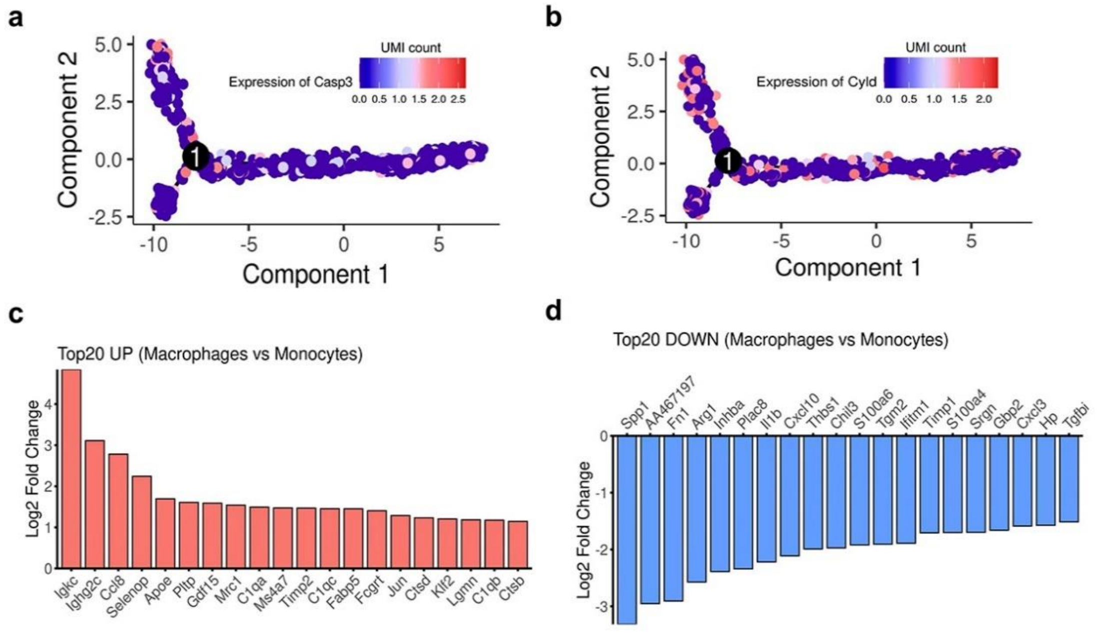

Supplementary Fig S5. The distribution and expression of cell death-related genes in monocytes and macrophages of ZIKV-infected testes.

(a, b) The signatures and expression dynamics of the expression of *Casp3* **(a)** and *Cyld* **(b)** in monocytes and macrophages were shown in pseudotime progression. **(c, d)** The top 20 upregulated genes **(c)** and the top 20 downregulated genes **(d)** during the differentiation from monocytes to macrophages.

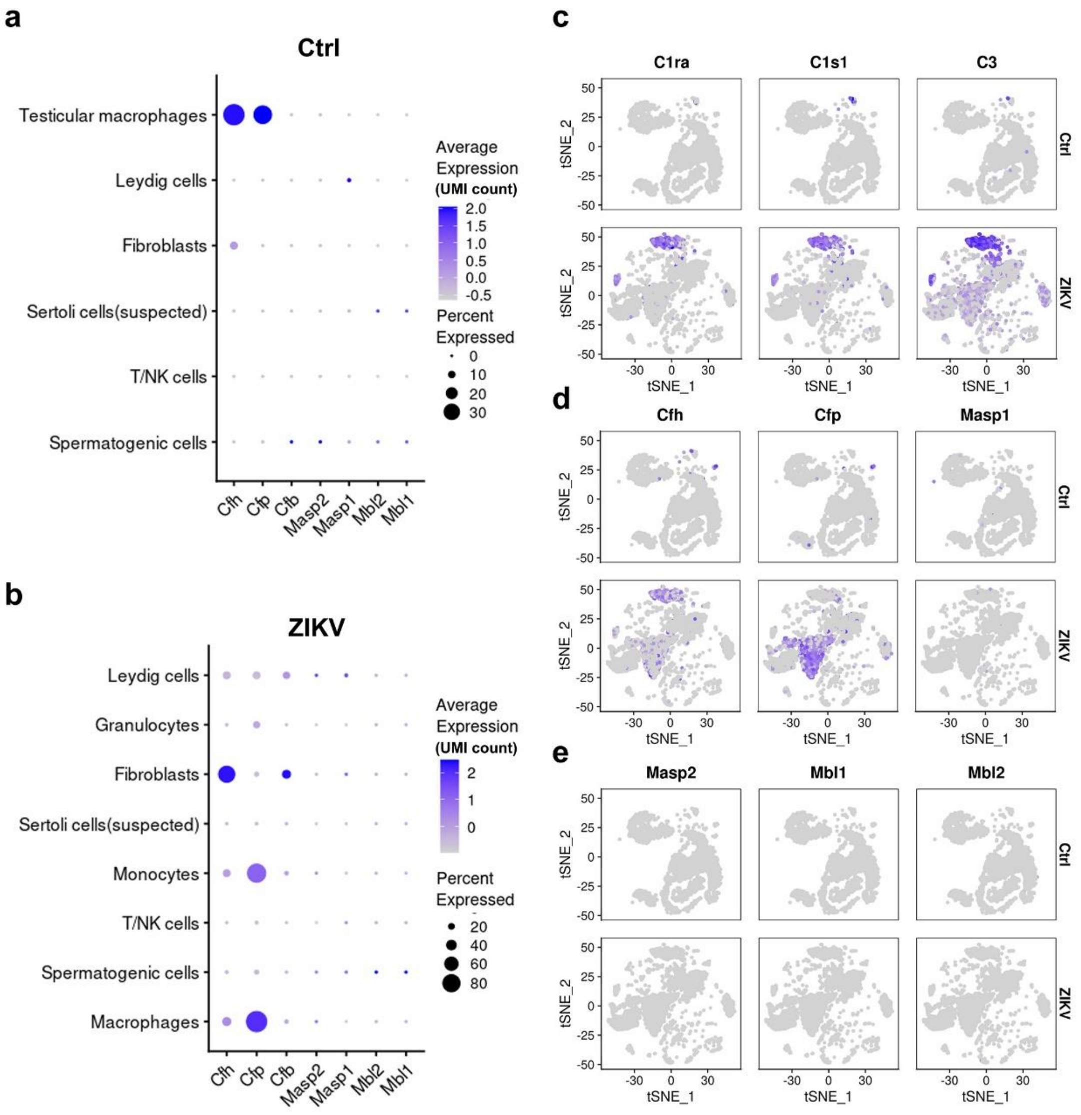

Supplementary Fig S6. The distribution and expression of complement components in ZIKV-infected testes.

(a, b) The expression of alternative/MBL complement activation related genes in all cell clusters from control testes **(a)** and ZIKV-infected testes at 14dpi **(b)**. **(c-e)** The distribution of cells expressing component genes as indicated was shown in tSNE chart.

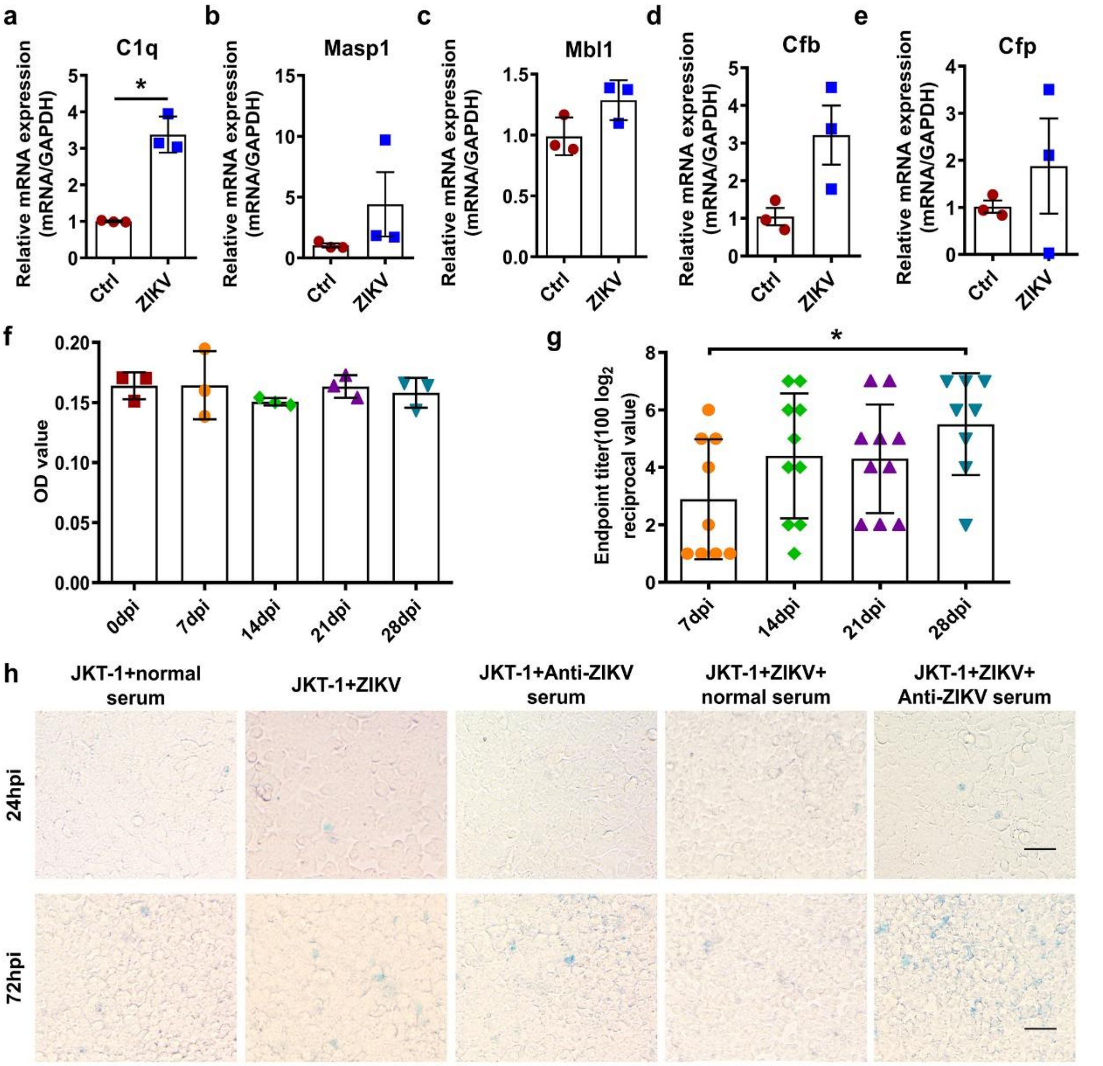

Supplementary Fig S7. ZIKV-infected spermatogenic cells could be damaged by ZIKV antigen-antibody complexes induced classical activation of complement system.

(a-e) The relative expression levels of complement components in testes from control or ZIKV-infected A6 male mice at 14dpi were analyzed by RT-PCR **(a-e)**. Results were shown as means ± SEM (n=3 mice for ctrl and 3 mice for ZIKV-infected) and analyzed using the two-sided Student's t test. *$p < 0.05$. **(f, g)** The titer of anti-sperm antibodies **(f, n=3 mice for each time point)**, or anti-ZIKV antibodies **(g, n=9, 10, 10 and 8 mice, respectively)** in serum of ZIKV-infected A6 male mice at 0-28dpi was measured by

ELISA, and analyzed using the one-way ANOVA. *p < 0.05. **(h)** Representative pictures of JKT-1 cells of in vitro complement activation assay. Scale bar 25 μm. Exact p values in Source Data file. Source data are provided as a Source Data file.

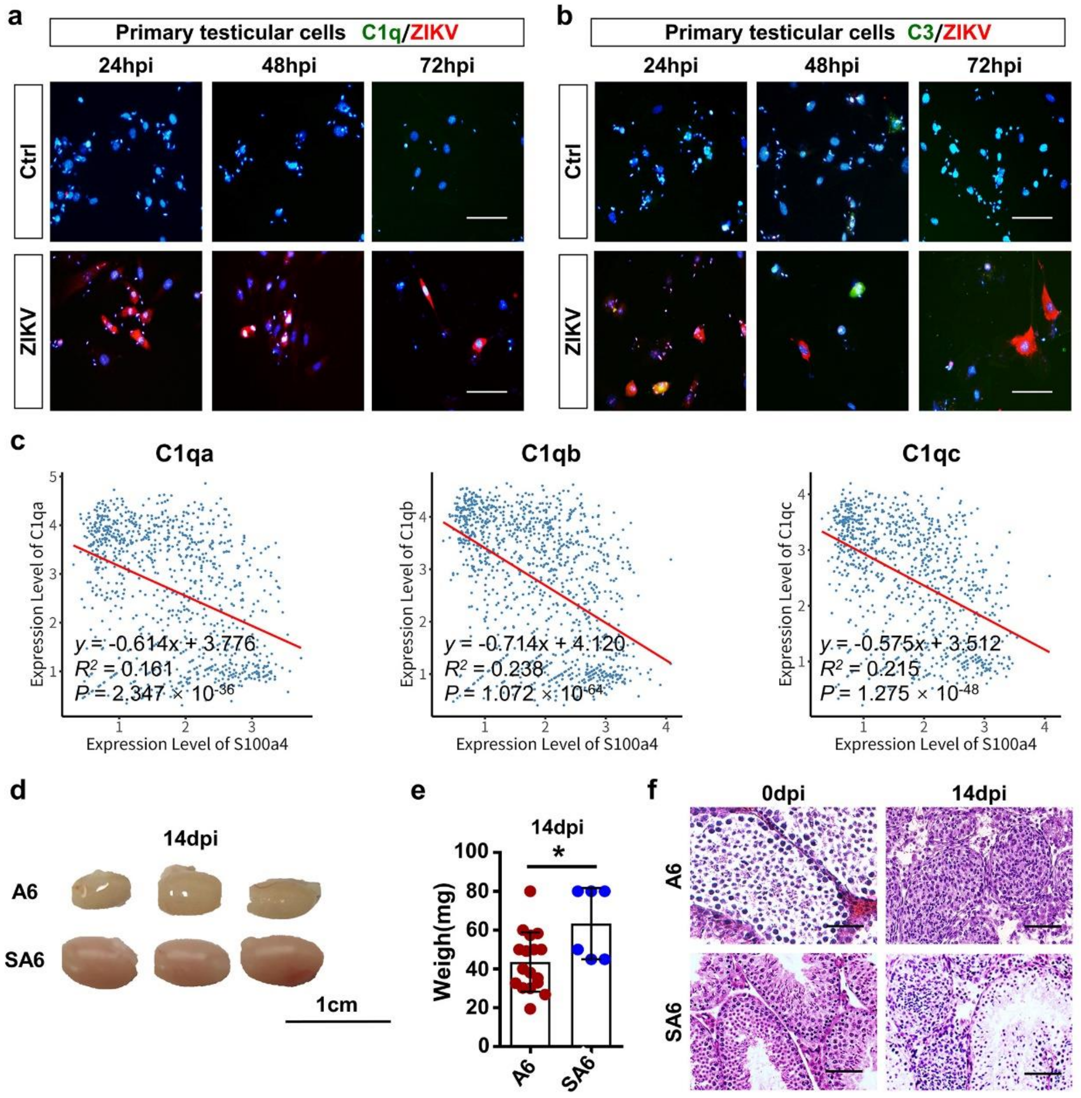

Supplementary Fig S8. *C1q* and *C3* were not expressed by testicular cells.

(a, b) The expression of *C1q* **(a)**, or *C3* **(b)** in control or ZIKV-infected primary testicular cells. Nuclei were stained with DAPI. Scale bar, 25 μm. **(c)** Negative correlation between the expression level of C1q genes and S100A4 gene in monocytes

and macrophages. Significance was examined by the two-sided t-test. **(d-f)** 6-8-week-old SA6 male mice were challenged with 1×10^4 ZIKV intraperitoneally. Representative pictures **(d)**, and weight **(e, n=17 testes for A6 and 6 testes for SA6)** of testes from ZIKV-infected A6 or SA6 male mice at 14dpi. Weight of ZIKV-infected testes were shown as means ± SEM and analyzed using the two-sided Student's t test. $^*p < 0.05$. Exact p values in Source Data file. HE staining **(f)** of testicular sections from ZIKV-infected A6 or SA6 male mice at 14dpi. Scale bar, 25 μm. Source data are provided as a Source Data file.

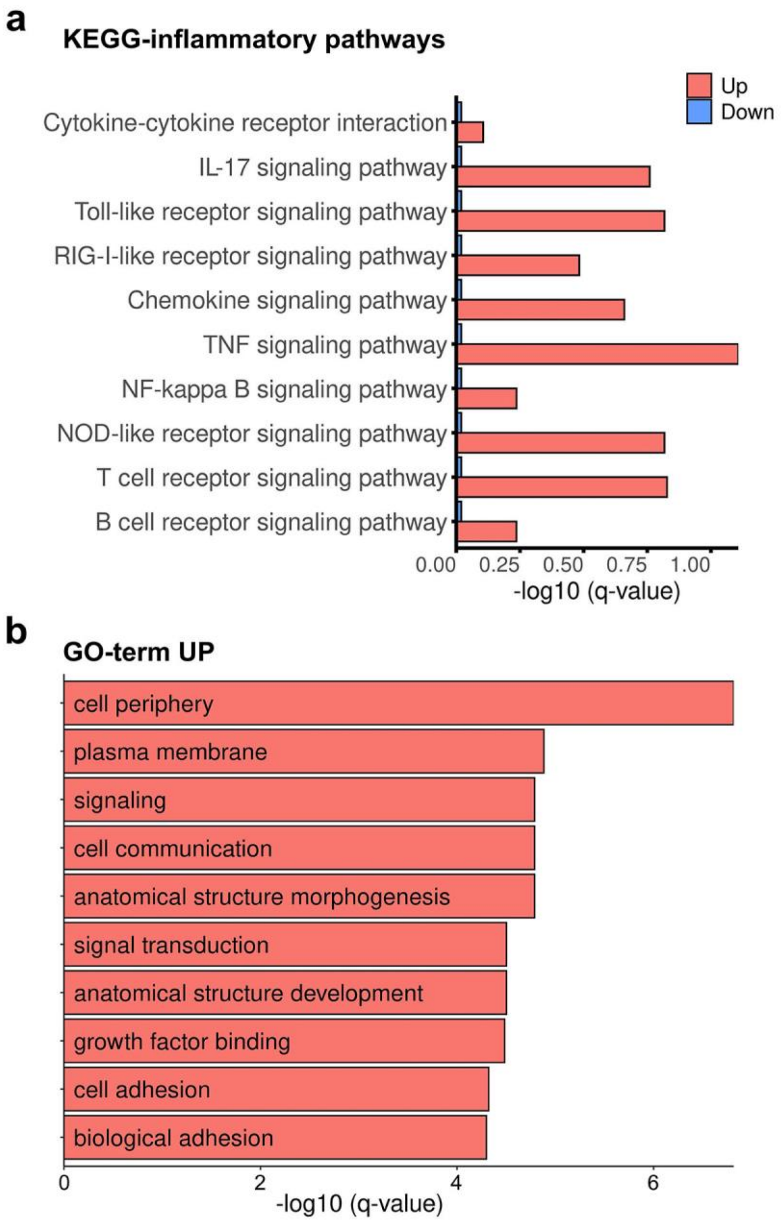

Supplementary Fig S9. The expression of and immune related genes in testes of ZIKV-infected *macaca leonine*.

Testes from uninfected or ZIKV-infected *macaca leonine* were isolated at 60dpi and subjected to RNA-sequencing. **(a)** KEGG enrichment analysis of multiple inflammatory pathways (n=3-4 *macaca leonine* for each group). **(b)** GO enrichment analysis of up-regulated mRNAs with top ten enrichment scores (n=3-4 *macaca leonine* for each group).

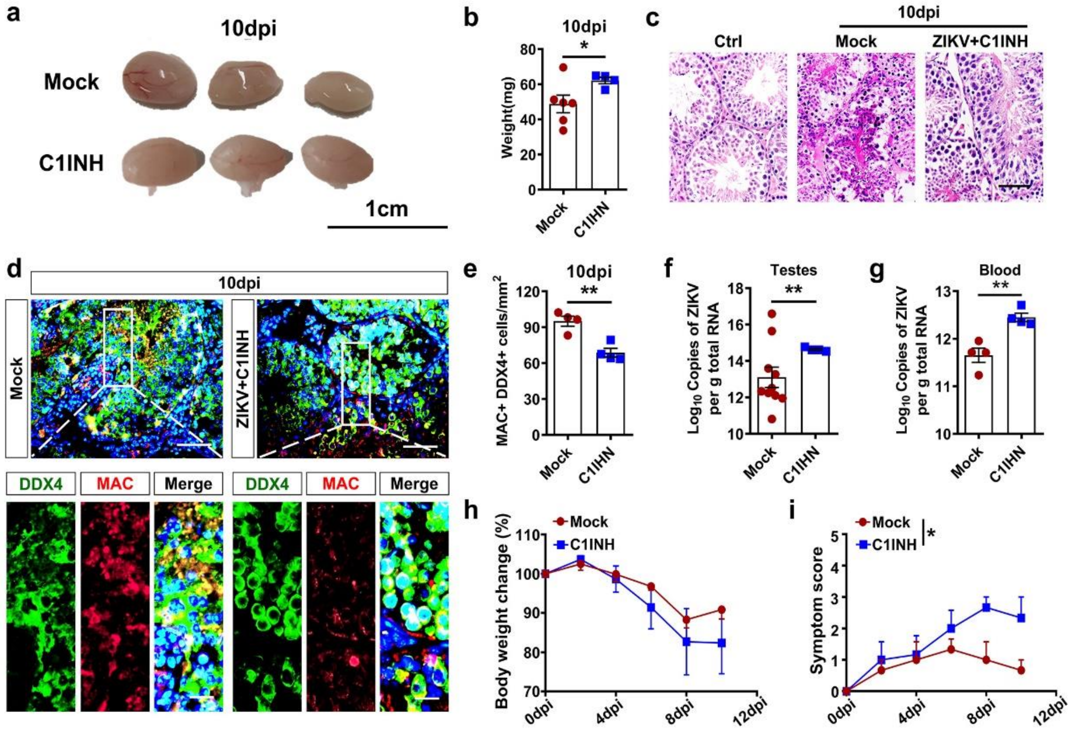

Supplementary Fig S10. C1INH alleviated ZIKV-induced spermatogenic cells damage

ZIKV-infected A6 male mice were intraperitoneally injected with 0.1 mg per mouse of C1 inhibitor (C1INH), or solvent alone (Mock), since 5dpi to euthanasia or death. **(a)** Representative pictures of testes in each group. **(b)** Testis weight at 10dpi. Results were shown as means ± SEM and analyzed using the two-sided Student's t test. * $p < 0.05$ (n= 6 testes for mock-infected and 4 testes for C1INH-treated). **(c)** HE staining of testicular sections at 10dpi. Scale bar, 25 μm. **(d, e)** The co-immunostaining of DDX4 and MAC **(d)**, in testes at 10dpi. MAC+ DDX4+ cells **(e)** were quantified. Results were shown as means ± SEM and expressed as cells/mm^2 and analyzed using the two-sided Student's t test. *$p < 0.05$, **$p < 0.01$ (n=4 mice for each group). Nuclei were stained with DAPI. Scale bar, 25 μm (10 μm in enlarged panels). **(f, g)** ZIKV RNA in testes **(f,**

n=6 testes for mock-infected and 4 testes for C1INH-treated) and whole blood **(g, n=4 mice for each group)** from ZIKV-infected A6 mice and C1INH-treated A6 mice at 10dpi were measured using RT-qPCR and shown as means ± SEM and analyzed using the two-sided Student's t test. *p < 0.05, **p < 0.01 (n= 4-6 mice for each group). **(h, i)** Body weights **(h, n=4 mice for mock-infected and 3 mice for C1INH-treated)** and symptom scores **(i, n=3 mice for each group)** were monitored daily. Results of body weight and symptom scores were shown as means ± SEM and the comparison of body weight and symptom scores between two groups were analyzed using repeated-measures ANOVA. *p < 0.05. Exact p values in Source Data file. Source data are provided as a Source Data file.

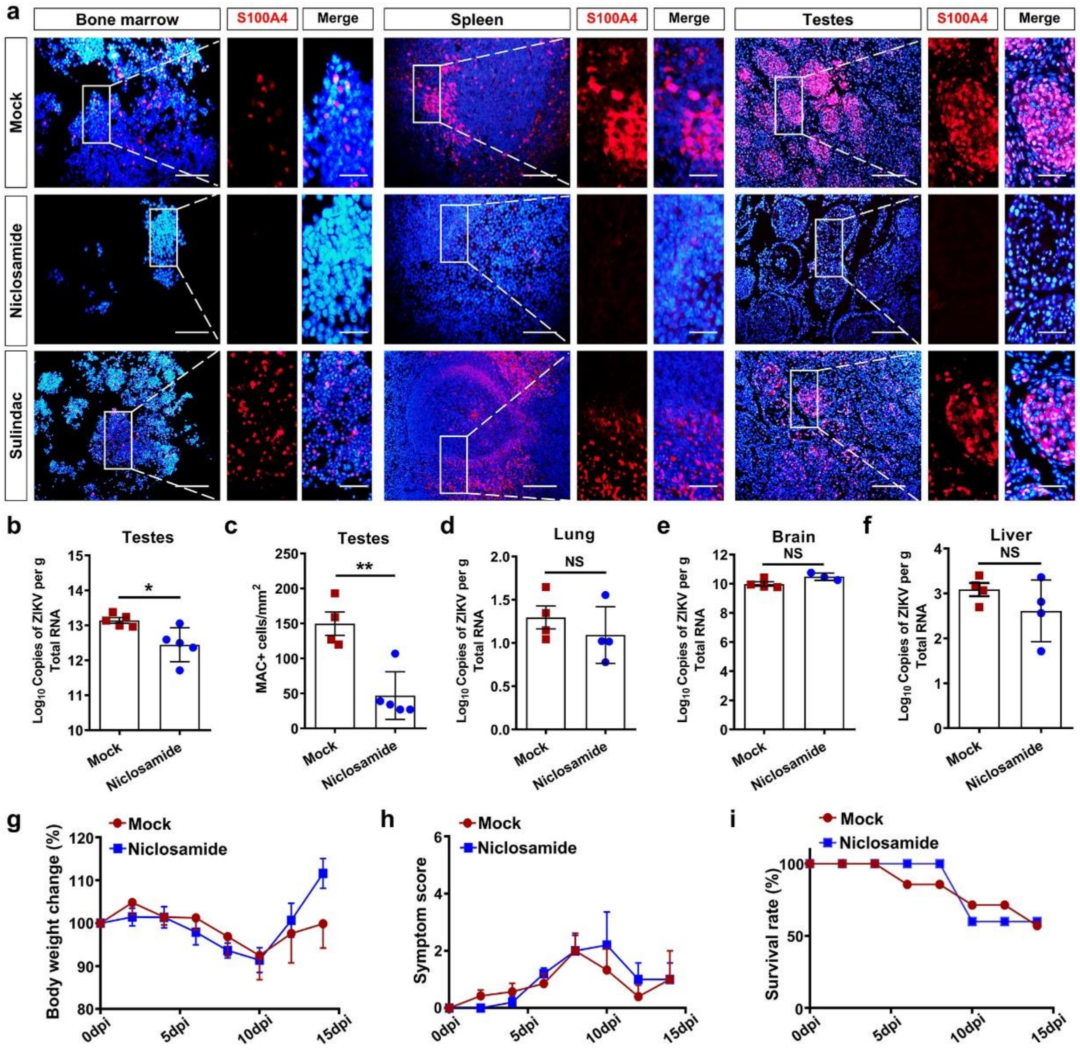

Supplementary Fig S11. S100A4 inhibitors niclosamide alleviated ZIKV-induced testicular damage.

ZIKV-infected A6 male mice were intraperitoneally injected with 20 mg/kg niclosamide or sulindac since 5dpi to euthanasia or death. Mock mice were injected with solvent alone. **(a)** The effects of S100A4 inhibitors on S100A4 expression in bone marrow, spleen and testis at 14dpi. Nuclei were shown with DAPI. Scale bar, 50 μm (20 μm in enlarged panels). **(b)** ZIKV RNA in testes from ZIKV-infected A6 mice and niclosamide-treated A6 mice at 14dpi were measured using RT-qPCR and shown as means ± SEM and analyzed using the two-sided Student's t test. $*p < 0.05$. (n= 5 mice

for each group). **(c)** MAC+ cells in testes from mock or niclosamide-treated A6 male mice were quantified. Results were shown as means ± SEM and expressed as cells/mm^2 and analyzed using the two-sided Student's t test. **p < 0.01. (n= 4 mice for mock-infected and 5 mice for niclosamide-treated) **(see Fig. 8e for representative pictures)**. **(d-f)** ZIKV RNA in lung, brain and liver from ZIKV-infected A6 mice and niclosamide-treated A6 mice at 14dpi were measured using RT-qPCR and shown as means ± SEM and analyzed using the two-sided Student's t test. *p < 0.05. (n= 4 mice for mock-infected and 3-4 mice for niclosamide-treated). **(g-i)** Body weights **(g, n=7 mice for mock-infected and 5 mice for niclosamide-treated)**, symptom scores **(h, n=7 mice for mock-infected and 5 mice for niclosamide-treated)** and survival rates **(i)** were monitored daily. Results of body weights and symptom scores were shown as means ± SEM and the comparison of body weight and symptom score between two groups were analyzed using repeated-measures ANOVA. Exact p values in Source Data file. Source data are provided as a Source Data file.

Gene name	P Value
Masp2	6.44E-07
C1s	0.001146
Cfp	0.276223
Masp1	0.634024
Cfi	0.704472
C1r	0.785686
C3	0.836768
Cd68	1.19E-06
Itgam/Cd11b	0.027609
Cd33	0.026016
Cd163	0.005772
S100a4	1
Mst1r	3.26E-05

**Mst1**	**0.000668**
**Macir**	**0.183525**
**Ddx4**	**0.970474**
**Sycp1**	**0.400584**
**Vim**	**0.151591**

Supplementary Table S1. P-value of differentially expressed genes in testes from ZIKV-infected *macaca leonine* at 60dpi.

P-value of 18 differentially expressed genes **(see Fig. 7j for representative pictures)** in testes from ZIKV-infected *macaca leonine* at 60dpi. Significance was examined by the two-sided exact test.
