# Supplementary material for: Single-cell RNA sequencing reveals the fragility of male spermatogenic cells to Zika virus-induced complement activation
Source: Nat Commun. 2023 Apr 29;14:2476. doi: 10.1038/s41467-023-38223-z (PMC10148584; doi:10.1038/s41467-023-38223-z)
Supplement: Supplementary file 3 — Reporting Summary [file 41467_2023_38223_MOESM3_ESM.pdf]

nature portfolio

Corresponding author(s): Jing An

Last updated by author(s): Apr 14, 2023

Reporting Summary

Nature Portfolio wishes to improve the reproducibility of the work that we publish. This form provides structure for consistency and transparency in reporting. For further information on Nature Portfolio policies, see our Editorial Policies and the Editorial Policy Checklist.

Statistics

For all statistical analyses, confirm that the following items are present in the figure legend, table legend, main text, or Methods section.

n/a	Confirmed	
☐	☒	The exact sample size (n) for each experimental group/condition, given as a discrete number and unit of measurement
☐	☒	A statement on whether measurements were taken from distinct samples or whether the same sample was measured repeatedly
☐	☒	The statistical test(s) used AND whether they are one- or two-sided<br>*Only common tests should be described solely by name; describe more complex techniques in the Methods section.*
☐	☒	A description of all covariates tested
☐	☒	A description of any assumptions or corrections, such as tests of normality and adjustment for multiple comparisons
☐	☒	A full description of the statistical parameters including central tendency (e.g. means) or other basic estimates (e.g. regression coefficient) AND variation (e.g. standard deviation) or associated estimates of uncertainty (e.g. confidence intervals)
☐	☒	For null hypothesis testing, the test statistic (e.g. F, t, r) with confidence intervals, effect sizes, degrees of freedom and P value noted<br>*Give P values as exact values whenever suitable.*
☒	☐	For Bayesian analysis, information on the choice of priors and Markov chain Monte Carlo settings
☒	☐	For hierarchical and complex designs, identification of the appropriate level for tests and full reporting of outcomes
☒	☐	Estimates of effect sizes (e.g. Cohen's d, Pearson's r), indicating how they were calculated

Our web collection on statistics for biologists contains articles on many of the points above.

Software and code

Policy information about availability of computer code

Data collection	All code associated with this manuscript have been uploaded to GitHub (https://github.com/anlab2022/ZIKV-testis-sc-data).
Data analysis	All statistical analysis were performed using SPSS 17.0 Software (IBM, Armonk, NY, USA) and rechecked by Microsoft Excel 2016. Positive staining cells in IFA assay or trypan blue positive cells mentioned above were analyzed with Image J v1.8.0.112 software

For manuscripts utilizing custom algorithms or software that are central to the research but not yet described in published literature, software must be made available to editors and reviewers. We strongly encourage code deposition in a community repository (e.g. GitHub). See the Nature Portfolio guidelines for submitting code & software for further information.

Data

Policy information about availability of data

All manuscripts must include a data availability statement. This statement should provide the following information, where applicable:

- Accession codes, unique identifiers, or web links for publicly available datasets
- A description of any restrictions on data availability
- For clinical datasets or third party data, please ensure that the statement adheres to our policy

All data needed to evaluate the conclusions in the paper are present in the paper and/or the Supplementary Materials. For read mapping of the RNA-Seq data from macaca leonine testes, the reference genome (Macaca_mulatta, Mmul_10, release-104) were downloaded from the Ensembl website. scRNA-seq data were aligned to Ensembl genome GRCm38 reference genome (release-95). The raw scRNA-seq data generated in this study has been deposited in the NCBI Sequence Read Archive under accession PRJNA756783 (https://www.ncbi.nlm.nih.gov/bioproject/PRJNA756783). The raw RNA-seq data generated in this study has been deposited

in the NCBI Sequence Read Archive under accession PRJNA756717 (https://www.ncbi.nlm.nih.gov/bioproject/?term=PRJNA756717). Source data are provided with this paper.

Human research participants

Policy information about studies involving human research participants and Sex and Gender in Research.

Reporting on sex and gender	n/a
Population characteristics	n/a
Recruitment	n/a
Ethics oversight	n/a

Note that full information on the approval of the study protocol must also be provided in the manuscript.

Field-specific reporting

Please select the one below that is the best fit for your research. If you are not sure, read the appropriate sections before making your selection.

- [x] Life sciences
- [ ] Behavioural & social sciences
- [ ] Ecological, evolutionary & environmental sciences

For a reference copy of the document with all sections, see nature.com/documents/nr-reporting-summary-flat.pdf

Life sciences study design

All studies must disclose on these points even when the disclosure is negative.

Sample size	Samples sizes were determined based on preliminary experiments.
Data exclusions	The animals died before measurement were excluded from the analysis. The criteria was pre-established.
Replication	Experimental findings were reliably reproduced in multiple (at least 3) independent experiments as indicated throughout the manuscript.
Randomization	All mice and macaca leonine were randomly allocated into different groups. Mice were also chosen randomly by genotype.
Blinding	The investigators (at least 3) were double-blinded during the counts of positive-staining cells. The criteria was pre-established. The data presented were the choices of the majority.

Reporting for specific materials, systems and methods

We require information from authors about some types of materials, experimental systems and methods used in many studies. Here, indicate whether each material, system or method listed is relevant to your study. If you are not sure if a list item applies to your research, read the appropriate section before selecting a response.

Materials & experimental systems

n/a	Involved in the study	
[ ]	[x]	Antibodies
[ ]	[x]	Eukaryotic cell lines
[x]	[ ]	Palaeontology and archaeology
[ ]	[x]	Animals and other organisms
[x]	[ ]	Clinical data
[x]	[ ]	Dual use research of concern

Methods

n/a	Involved in the study	
[x]	[ ]	ChIP-seq
[x]	[ ]	Flow cytometry
[x]	[ ]	MRI-based neuroimaging

Antibodies

Antibodies used	1. rat anti-mouse C1q antibody (1:100, Abcam, ab11861), 2. rat anti-mouse C3 antibody (1:100, Abcam, ab11862), 3. rabbit anti-mouse membrane attack complex (MAC) antibody (1:100, Abcam, ab55811), 4. rabbit anti-mouse CD45 antibody (1:100, Abcam, ab10558), 5. rabbit anti-mouse Granzyme B antibody (GZMB) (1:100, Abcam, ab4059), 6. rabbit anti-mouse F4/80 antibody (1:100, Abcam, ab111101), 7. rabbit anti-mouse Caspase-9 antibody (1:500, Abcam, ab202068), 8. rabbit anti-mouse Caspase-8 antibody (1:500, Abcam, ab227430), 9. rabbit anti-mouse cleaved Caspase-3 antibody (1:500, Cell Signaling Technology, 9664S), 10. rabbit anti-mouse Caspase-1 antibody (1:500, Abcam, ab74279), 11. mouse anti-mouse DDX4 antibody (1:100, Abcam, ab27591), 12. rabbit

anti-mouse DDX4 antibody (Abcam, ab13840), 13. rabbit anti-mouse S100A4 antibody (1:500, Cell Signaling Technology, 13018S), 14. mouse anti-ZIKV antibody 4G2 (anti-ZIKV E protein antibody, preparate and preserve by our group), 15. donkey anti-mouse IgG (1:1000, Alexa Fluor R 488, A21202, Life technologies), 16. donkey anti-rabbit IgG (1:1000, Alexa Fluor R 594, A21207, Life technologies), 17. goat anti-rabbit IgG (1:1000, Alexa Fluor 488, A-11008, Life technologies), 18. goat anti-mouse IgG 594 (1:1000, Alexa Fluor, A-11005, Life technologies), 19. goat anti-rat IgG (1:1000, Alexa Fluor 488, ab150157), 20. goat anti-mouse HRP-labeled secondary antibodies (1/100000, Abcam, ab6789)

Validation

1. rat anti-mouse C1q antibody (Abcam, ab11861),
Validated in IHC-Fr and tested in Mouse. Referenced in 12 publications. https://www.abcam.cn/c1q-antibody-7h8-ab11861.html
2. rat anti-mouse C3 antibody (Abcam, ab11862),
Validated in ICC/IF and tested in Mouse. Referenced in 38 publications. https://www.abcam.cn/c3-antibody-11h9-ab11862.html
3. rabbit anti-mouse membrane attack complex (MAC) antibody (Abcam, ab55811),
Validated in IHC-P, ICC/IF and tested in Mouse, Human. Referenced in 59 publications. https://www.abcam.cn/c5b-9-antibody-ab55811.html
4. rabbit anti-mouse CD45 antibody (Abcam, ab10558),
Validated in Flow Cyt, IHC-FoFr, WB, IHC-P and tested in Mouse, Rat, Human. Referenced in 258 publications. https://www.abcam.cn/cd45-antibody-ab10558.html
5. rabbit anti-mouse Granzyme B antibody (GZMB) (Abcam, ab4059),
Validated in IHC-P and tested in Mouse, Human. Referenced in 107 publications. https://www.abcam.cn/granzyme-b-antibody-ab4059.html
6. rabbit anti-mouse F4/80 antibody (Abcam, ab111101),
Validated in IHC-P and tested in Mouse. Referenced in 86 publications. https://www.abcam.cn/f480-antibody-sp115-ab111101.html
7. rabbit anti-mouse Caspase-9 antibody (Abcam, ab202068),
Validated in IHC-P, WB, ICC/IF, IP and tested in Mouse, Human. Referenced in 72 publications. https://www.abcam.cn/caspase-9-antibody-epr18107-ab202068.html
8. rabbit anti-mouse Caspase-8 antibody (Abcam, ab227430),
Validated in WB, IHC-P, ICC/IF and tested in Mouse, Rat, Human. Referenced in 7 publications. https://www.abcam.cn/caspase-8-antibody-ab227430.html
9. rabbit anti-mouse cleaved Caspase-3 antibody (Cell Signaling Technology, 9664S),
Validated in WB, IHC-P, ICC/IF and tested in Human, Mouse, Rat, Monkey. Referenced in 3226 publications. https://www.cellsignal.com/products/primary-antibodies/cleaved-caspase-3-asp175-5a1e-rabbit-mab/9664
10. rabbit anti-mouse Caspase-1 antibody (Abcam, ab74279),
Validated in IHC-P and tested in Human, Mouse. Referenced in 5 publications. https://www.abcam.cn/caspase-1-antibody-ab74279.html
11. mouse anti-mouse DDX4 antibody (Abcam, ab27591),
Validated in WB, IHC-P, ICC/IF and tested in Human, Mouse. Referenced in 57 publications. https://www.abcam.cn/ddx4--mvh-antibody-mabcam27591-ab27591.html
12. rabbit anti-mouse DDX4 antibody (Abcam, ab13840),
Validated in WB, IHC-P, IHC-Fr and tested in Human, Mouse. Referenced in 388 publications. https://www.abcam.cn/ddx4--mvh-antibody-ab13840.html
13. rabbit anti-mouse S100A4 antibody (Cell Signaling Technology, 13018S),
Validated in WB, IHC-P and tested in Human, Mouse. Referenced in 20 publications. https://www.cellsignal.com/products/primary-antibodies/s100a4-d9f9d-rabbit-mab/13018?site-search-type=Products&N=4294956287&Ntt=13018s&fromPage=plp&_requestid=209242
14. mouse anti-ZIKV antibody 4G2 (anti-ZIKV E protein antibody) were validated in our previous paper (pmid: 35714850, 36241087).

Eukaryotic cell lines

Policy information about cell lines and Sex and Gender in Research

Cell line source(s)

1. The Vero cell line was purchased from National Infrastructure of Cell Line Resource, China.
2. The C6/36 cell line was purchased from National Infrastructure of Cell Line Resource, China.
3. The JKT-1 cell line was purchased from Mlbio, China.

Authentication

1. The Vero cell line has been authenticated using STR by National Infrastructure of Cell Line Resource, China.
2. The C6/36 cell line has been authenticated using STR by National Infrastructure of Cell Line Resource, China.
3. The JKT-1 cell line has been authenticated using STR by Mlbio, China.

Mycoplasma contamination

All cell lines in this study were not contaminated with mycoplasma.

Commonly misidentified lines
(See ICLAC register)

No commonly misidentified cell line was used in this research.

Animals and other research organisms

Policy information about studies involving animals; ARRIVE guidelines recommended for reporting animal research, and Sex and Gender in Research

Laboratory animals

A6 mice were purchased from the Institute of Zoology, Chinese Academy of Medical Sciences. Balb/c mice and SA6 mice were kept in our laboratory. hSTAT2 KI mice were kindly provided by the Jackson Laboratory. The mice were maintained in a specific pathogen-free animal facility. 6-8-week-old male mice, including A6, SA6 and hSTAT2 KI mice, were used for mouse study. All mice were fed

	standard chow (irradiated cobalt-60) and housed in groups of up to five. Lighting was time controlled on a standard condition (12/12 h light/dark cycle). Temperature and humidity were stable and consistent at 20–26 °C and 40–70%, respectively.<br>Macaca leonine (northern pigtailed macaques) were kindly provide by Kunming Institute of Zoology, macaca leonines were also maintained in a specific pathogen-free animal facility. Five-year-old adult male macaca leonine were used for this study.
Wild animals	n/a. This study did not involve wild animals.
Reporting on sex	All experiments were conducted on male mice or male macaca leonine.
Field-collected samples	n/a. This study did not involve samples collected from the field.
Ethics oversight	All the animal experimental protocols were approved by and conducted in accordance with the guidelines established by the Institutional Animal Care and the Animal Ethics Committees of Capital Medical University, Beijing, China.

Note that full information on the approval of the study protocol must also be provided in the manuscript.
